# The Role of hCG and Histamine in Emesis Gravidarum and Use of a Chewing Gum Containing Vitamin C as a Treatment Option: A Double-Blinded, Randomized, Controlled Trial

**DOI:** 10.3390/jcm13175099

**Published:** 2024-08-28

**Authors:** Philipp Foessleitner, Lilly Rager, Fanny Mikula, Marlene Hager, Sonja Granser, Helmuth Haslacher, Jonas Brugger, Alex Farr

**Affiliations:** 1 Division of Obstetrics and Feto-Maternal Medicine, Department of Obstetrics and Gynecology, Medical University of Vienna, A-1090 Vienna, Austria; philipp.foessleitner@meduniwien.ac.at (P.F.); fanny.mikula@meduniwien.ac.at (F.M.); sonja.granser@meduniwien.ac.at (S.G.); 2 Vincent Center for Reproductive Biology, Massachusetts General Hospital, Harvard Medical School, Boston, MA 02114, USA; 3 Clinical Division of Gynecological Endocrinology and Reproductive Medicine, Department of Obstetrics and Gynecology, Medical University of Vienna, A-1090 Vienna, Austria; marlene.hager@meduniwien.ac.at; 4 Department of Laboratory Medicine, Medical University of Vienna, A-1090 Vienna, Austria; helmuth.haslacher@meduniwien.ac.at; 5 Center for Medical Data Science, Informatics and Intelligent Systems, Medical University of Vienna, A-1090 Vienna, Austria; jonas.brugger@meduniwien.ac.at

**Keywords:** emesis gravidarum, histamine, human chorionic gonadotropin, nausea, vomiting, pregnancy complications, vitamin C, diamine oxidase

## Abstract

**Background:** Nausea and vomiting in pregnancy (NVP), or emesis gravidarum, is a frequent complication of early gestation with unclear causes, suspected to involve genetic, hormonal, and gastrointestinal factors. Our study investigated the association of human chorionic gonadotropin (hCG), histamine, diamine oxidase (DAO), thyroxine and pyridoxine and the severity of NVP symptoms and assessed the efficacy of a vitamin C-containing chewing gum as a potential NVP treatment. **Methods:** In this prospective, double-blinded, randomized, controlled trial, 111 participants were assigned to receive vitamin C-containing chewing gum, placebo gum, or no treatment at two follow-ups during early pregnancy. Maternal serum levels of hCG, histamine, DAO, thyroxine, and pyridoxine were measured and correlated with NVP severity using the Pregnancy-Unique Quantification of Emesis and Nausea (PUQE-24) score. **Results:** Elevated maternal hCG levels were significantly associated with an increased PUQE-24 score (*p* < 0.001), while histamine levels showed no significant correlation (*p* = 0.68). Maternal DAO levels negatively correlated with NVP symptoms (*p* < 0.001) and elevated thyroxine (*p* < 0.001) and pyridoxine levels (*p* < 0.001) were associated with increased PUQE-24 scores. The vitamin C-containing chewing gum did not demonstrate efficacy in alleviating NVP symptoms compared to placebo gum or no treatment during the first (*p* = 0.62) and second follow-up visits (*p* = 0.87). **Conclusions:** Our study underscores the complexity of factors contributing to NVP, highlighting the significant roles of hCG and DAO, while histamine levels appear unrelated. Maternal thyroxine and pyridoxine levels also significantly correlate with NVP symptoms. Vitamin C-containing chewing gum was not effective as a treatment for NVP. Further large-scale studies are needed to better understand these interactions and develop targeted treatments in the future.

## 1. Introduction

Nausea and vomiting in pregnancy (NVP), also known as emesis gravidarum, is a common complication in early pregnancy, affecting 70–80% of pregnant women, with symptoms typically beginning around 2 to 4 gestational weeks and peaking between 9 to 16 weeks [1]. The broadly used term “morning sickness” is misleading, since symptoms occur throughout the day in up to 98% of affected women [2]. In about 10% of pregnancies, NVP persists until term, and 0.3 to 2% of women suffer from hyperemesis gravidarum (HG), which can lead to dehydration and electrolyte imbalance, often requiring hospitalization [1,2]. Despite often being perceived as a typical discomfort of early pregnancy, NVP is associated with an increased risk for serious health disorders, such as arterial hypertension, pre-eclampsia and depression [3,4].

Up to date, the etiology of emesis gravidarum remains unclear, though it is believed to be multifactorial, involving genetic, endocrine, and gastrointestinal factors [1]. Thereby, NVP appears to be related to the placenta rather than the fetus, as evidenced by cases of complete hydatidiform mole, where significant nausea occurs despite the absence of fetal development [5].

Human chorionic gonadotropin (hCG) has been implicated as a potential causal factor, with studies showing a correlation between nausea severity and maternal serum hCG levels [5,6]. Additionally, pregnancies with higher hCG levels such as twins or hydatidiform mole are associated with an increased risk of suffering from nausea and vomiting. Given the established association between estrogen, progesterone and nausea, it is hypothesized that the stimulatory impact of hCG on estrogen and progesterone production may serve as the underlying mechanism [5].

Since anti-histaminic treatments show efficacy in reducing NVP symptoms [5], elevated histamine levels emerge as another possible contributing factor emesis gravidarum [7]. During pregnancy, histamine is mainly produced by the mast cells in the endometrium, myometrium, placenta and decidua [7]. High expressions of the histamine-producing enzyme histidine decarboxylase in the placenta and an increased number of histamine receptors in the decidua suggest a physiological role of histamine during pregnancy. Acting as a counterpart, diamine oxidase (DAO) degrades histamine by oxidative deamination and thereby decreases histamine blood levels [8]. DAO is produced in the decidua and the trophoblast and acts as a barrier to stop excessive transport of histamine into the maternal and fetal circulation [7]. DAO levels increase rapidly during the first 20–22 weeks of gestation, possibly in response to high histamine levels, achieving a balance that coincides with the resolution of NVP symptoms in most pregnancies [7,9,10].

Additionally, hCG stimulates the thyroid gland due to its similarity to thyrotropin (TSH), leading to reduced TSH and elevated free thyroxine (fT4) levels, a condition that usually normalizes around the 20th week of gestation [5,11,12]. The question of whether or not this temporary hyperthyroidism is linked to emesis gravidarum remains controversial [11].

When it comes to the potential treatment options for NVP, research has proven the capacity of vitamin C to reduce the circulating histamine levels, both in allergic and non-allergic contexts, and has been used to treat histamine intolerance [13,14]. Additionally, oral vitamin C intake proved successful in reducing the symptom of nausea due to seasickness [14]. Chewing gum has emerged as an appropriate route of administration that seems to be comparable to ondansetron for managing postoperative nausea and vomiting [15].

In light of the existing literature, this study aimed to evaluate a possible association between maternal hCG or histamine levels and the severity of nausea and vomiting in early pregnancy and to assess the efficacy of a vitamin C-containing chewing gum in reducing symptoms of emesis gravidarum as a new treatment option for NVP. As a secondary aim, we were investigating the impact of maternal thyroxine and pyridoxine levels on the clinical course of NVP.

## 2. Material and Methods

### 2.1. Ethics Statement

The presented study was approved by the ethics committee of the Medical University of Vienna (application number: 1949/2019) and registered at clinicaltrials.gov (Clinicaltrials-ID: NCT04284696); it was performed in accordance with the Declaration of Helsinki and Good Scientific Practice guidelines, following the CONSORT guidelines for parallel group randomized trials [16]. An informed consent was obtained from all participants prior to study inclusion. All patient records were pseudo-anonymized and de-identified prior to the analysis.

### 2.2. Setting and Study Population

This prospective, randomized, double-blinded, placebo-controlled trial was conducted at the Medical University of Vienna, Department of Obstetrics and Gynecology, from February 2020 to March 2022. Our tertiary obstetric center is specialized on the service of high-risk pregnancy care, providing clinical services and consultations for approximately 2800 deliveries per year, including referrals from central and eastern Europe. Study inclusion was offered to all women 18 years or older with a singleton pregnancy, presenting for birth registration during the first trimester at our department, experiencing symptoms of NVP, and not having undergone antiemetic treatment previously.

### 2.3. Study Procedure

At study inclusion, participants were randomized to one of the following 3 groups:

- Participants suffering from emesis gravidarum receiving 150 mg vitamin C-containing chewing gums (verum) “ad libitum” daily for 2 weeks (defined as the vitamin C group).
- Participants suffering from emesis gravidarum receiving chewing gums without an active agent (placebo) “ad libitum” daily for 2 weeks (defined as the placebo group).
- Participants suffering from emesis gravidarum receiving no treatment (defined as the no treatment group).

Randomization was carried out by means of the GCP-compliant web-based randomizer (Medical University of Graz, Austria). The ratio of the allocation to the 3 groups was 1:1:1. Smoking was included as a co-variate in the randomization, since nicotine abuse correlates with a lower risk of suffering from (hyper-)emesis gravidarum [17,18]. Investigators and participants were blinded in regard to the vitamin C and placebo group (verum vs. placebo). If allocated to the vitamin C or placebo group, participants received a badge of chewing gums to take “ad libitum” for the following 2 weeks. Patients were instructed to take two chewing gums in the morning at the onset of nausea and then “ad libitum” additional chewing gums throughout the day when needed, up to a maximum of eight gums (1200 mg vitamin C for the vitamin C group). Both chewing gums were manufactured by Frey AG (Migros-Genossenschafts-Bund, Zurich, Switzerland) and were not distinguishable from each other in taste or appearance. The difference between verum and placebo was solely the content of 150 mg vitamin C per chewing gum (verum) or 0 mg of vitamin C (placebo). Other ingredients identical in both verum and placebo gum were as follows: sweeteners isomalt, sorbitol, maltisirup, sucralose, acesulfame K, chewing gum (with antioxidant E306), sodium L-ascorbate, flavorings, acidifier (apple and citric acid), thickener gum arabic, dye E171, humectant E422 and E1518, coating agents E903 and E553b. The intake of any antiemetic medication was prohibited during the study period for all groups.

Upon inclusion in the study, participants were requested to complete a validated questionnaire, the modified Pregnancy-Unique Quantification of Emesis and Nausea (PUQE-24) score [19,20,21], for the assessment of the severity of emesis gravidarum. Furthermore, demographic data were collected using a case-report-form. The questionnaire is provided in Figure S1 for reference. Furthermore, venous blood samples were obtained through phlebotomy to assess levels of beta subunit-hCG, histamine, DAO, thyroxine, and pyridoxine in maternal circulation. Subsequent evaluations were conducted at two follow-up visits: firstly, approximately 2 weeks post-enrollment, predominantly coinciding with routine nuchal translucency screening (performed between 11 and 14 weeks of gestation); and secondly, during routine organ scanning (performed between 20 and 24 weeks of gestation), or alternatively, at least 2 weeks following the first follow-up. These follow-up assessments encompassed a re-evaluation of the participant’s current condition using the modified PUQE-24 score, along with a repetition of blood sampling procedures. Additionally, the quantity of chewing gums consumed was determined by the collection and counting of used packs during the first follow-up visit. The precise study procedure is shown in Figure S2.

### 2.4. Determination of the Degree of Nausea and Vomiting

As previously described, emesis gravidarum was determined at each study visit using the modified PUQE-24 score [19,20,21]. Thereby, patients were asked to complete 3 questions regarding the timespans of nausea, vomiting and retching within the last 24 h. As mentioned above, the questionnaire is provided in Figure S1. Each response was assigned a value from one (no symptoms) to five points (severe symptoms). From these values, a cumulative score was calculated to determine the severity of emesis gravidarum: scores of ≤3 points indicated no NVP, 4–6 points was classified as mild NVP, 7–12 points signified moderate symptoms, while scores of ≥13 points indicated severe NVP. Only patients who had a PUQE-24 score of 4 or more points were included in the study.

### 2.5. Data Collection and Statistical Analysis

Data collection was performed using the case report forms (CRFs) obtained at study inclusion and the obstetric documentation system PIA Fetal Database, version 5.6.28.56 (General Electric Company, GE Viewpoint, Munich, Germany) as well as the hospital information system of the hospital (SAP SE Inc., Walldorf, Germany). Power analysis was conducted prior to the start of the study. We assumed a standard deviation for the PUQE-24 score of 3 points and a mean difference of 2 points between the vitamin C group and, both, placebo and no treatment groups and zero mean difference between placebo and no treatment groups. Under these assumptions a total sample size of 102 is required to achieve 80% power at the 5% significance level with an F-Test for the null hypothesis of no mean differences between the three groups. The final planned sample size was 126 patients to allow for up to eight drop-outs (20%) per group. For the primary analysis, we used linear models with the PUQE-24 score at the first and second follow-up as the outcome and the vitamin C group as the predictor, adjusting for the baseline PUQE-24 score and follow-up week. The null hypothesis of no mean differences between the study groups was tested from the linear model using an F-test. Post hoc comparisons between groups were planned if the F-test was significant. Changes between visits in the overall study population as well as in each study group separately were assessed using paired *t*-tests. Spearman correlation coefficients were employed to assess the association between the number of chewed gums and the PUQE-24 score, restricted to the two gum-receiving groups. The same analyses were conducted using the PUQE-24 score at the second follow-up. To investigate the association of the PUQE-24 Score and maternal serum levels of hCG, histamine, DAO, thyroxine and pyridoxine, we calculated the global within-subject correlation for repeated measurements using the Bland–Altman method [22]. Correlation coefficients r ≥ 0.10 were graded as weak, r ≥ 0.30 as moderate, and r ≥ 0.50 as strong correlation. Descriptive statistics were calculated for all variables of interest. For continuous variables, mean and standard deviation are presented unless otherwise specified. Ordinally scaled and binary variables are presented with a number and percentage. The significance level was set to α = 0.05. Statistical analysis was performed using R, version 4.2.0 or higher.

## 3. Results

### 3.1. Study Participants and Baseline Characteristics

Out of the total cohort of 126 pregnant women, 15 had to subsequently be excluded during the first or second follow-up visits due to miscarriage (*n* = 7), non-attendance at the follow-up visits (*n* = 4), or voluntary withdrawal from the study (*n* = 4). Among the remaining 111 participants, 41 were randomized to the vitamin C group, 35 to the placebo group, and 35 to the no treatment group. The study inclusion is depicted in Figure S3. Mean age at the time of study inclusion was 33.2 years (standard deviation, ±6.1) for participants in the vitamin C group, 32.2 years (±4.9) for those in the placebo group, and 32.2 years (±5.2) for individuals in the no treatment group (*p* = 0.65). Women were enrolled at a median of 10 [interquartile range, 9–11] gestational weeks (*p* = 0.85 between the groups). Symptoms of NVP commenced during 5.3 weeks (±1.6) on average in the vitamin C group, 6.0 weeks (±1.9) in the placebo group, and 5.5 weeks (±1.1) in the no treatment group (*p* = 0.15). The initial median PUQE-24 score at study inclusion was 8 [7–10] in the vitamin C group, 9 [7–10] in the placebo group, and 8 [7–9] points in the no treatment group (*p* = 0.25). All other baseline characteristics, including nicotine use, were homogenously distributed across the study population and are reported in Table 1.

### 3.2. Efficacy of a Vitamin C-Containing Chewing Gum in Reducing Symptoms of NVP

**Figure 1:**
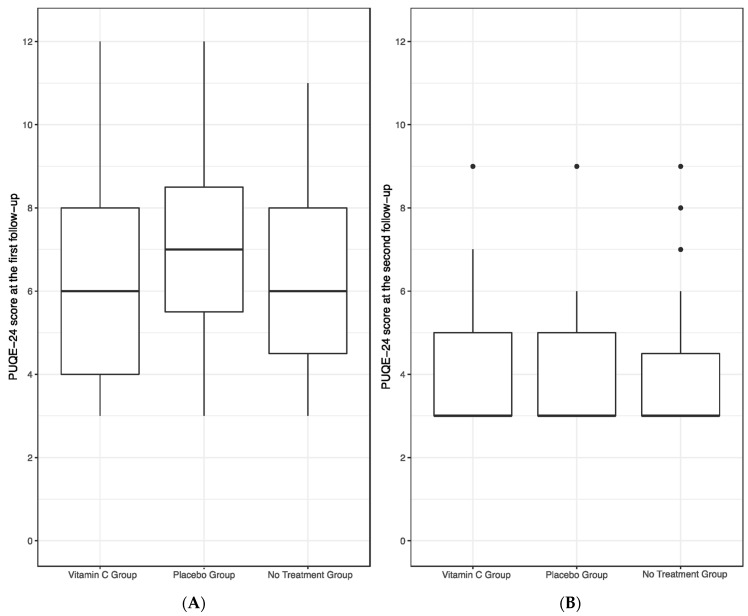
PUQE-24 score of the vitamin C, placebo and no treatment group at the first (**A**) and second follow-up (**B**). No significant differences in PUQE-24 scores were found between the groups at both follow-up visits, as assessed by a linear model with the vitamin C group as the main predictor and PUQE-24 scores as well as follow-up week of gestation as co-variates.

The first follow-up visit served as the primary endpoint to evaluate the efficacy of a vitamin C-containing chewing gum in reducing the symptoms of NVP. Statistical analysis revealed no significant differences in the PUQE-24 scores between the vitamin C, placebo and no treatment group (*p* = 0.62). Results are graphically presented in Figure 1A. Notably, there was no significant correlation between the quantity of chewing gums consumed and the PUQE-24 score within both the vitamin C and placebo groups (*p* = 0.19).

Subsequently, the second follow-up visit served as our secondary endpoint in assessing the efficacy of chewing gum. No statistically significant differences were observed among the vitamin C, placebo, and no treatment groups in terms of PUQE-24 scores (*p* = 0.87), as illustrated in Figure 1B. Additionally, also during the second follow-up, no significant correlation emerged between the quantity of chewing gums consumed and the PUQE-24 score within both the vitamin C and placebo groups (*p* = 0.77). No patient reported any adverse effect of the chewing gums.

The PUQE-24 score significantly decreased from baseline to the first follow-up visit and furthermore to the second follow-up visit in the overall study population and in each study group separately (all with *p* < 0.001).

### 3.3. Correlation of hCG and Histamine with Symptoms of NVP

**Figure 2:**
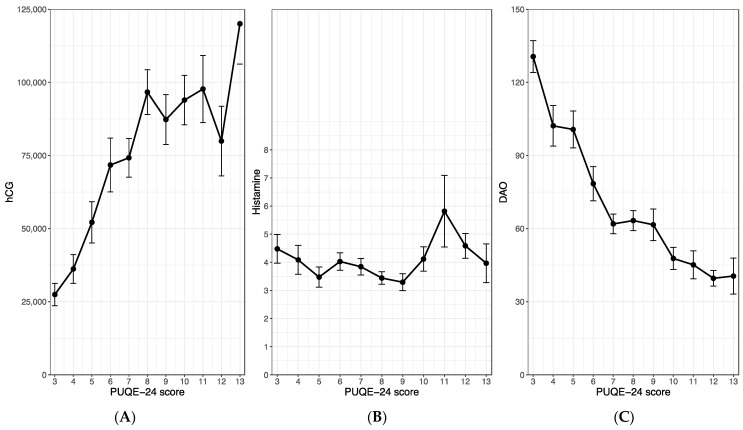
Correlations between the PUQE-24 score and the maternal serum levels of hCG (**A**), histamine (**B**) and diamine oxidase (**C**). The figures show the mean and standard error of the maternal serum levels with regard to the observed PUQE-score values. All three study groups were merged for this analysis. hCG, human chorionic gonadotropin.

We evaluated a potential correlation between maternal hCG or histamine levels and the severity of nausea and vomiting in early pregnancy, quantified by the PUQE-24 score, using the within-subject correlation analysis of Bland and Altman [22]. We found a highly significant and strongly positive correlation between maternal hCG serum levels and the PUQE-24 score (r = 0.70; *p* < 0.001), as shown in Figure 2A. While our analysis did not unveil any association between maternal histamine levels and the symptoms of NVP (r = −0.03, *p* = 0.68; Figure 2B), interestingly, we observed a significant and strongly negative correlation between the histamine-degrading enzyme, DAO, and the PUQE-24 score (r = −0.70, *p* < 0.001; Figure 2C). Furthermore, no significant association emerged between maternal histamine and DAO levels (r = 0.05; *p* = 0.49).

### 3.4. Influence of Thyroxine and Vitamin B6 Levels on NVP

**Figure 3:**
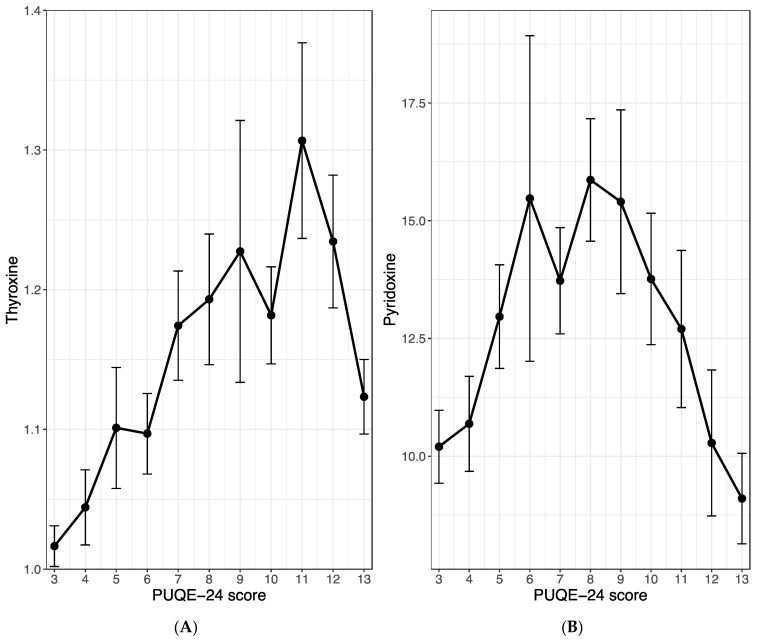
Correlation between the PUQE-24 score and the maternal serum level of thyroxine (**A**) and pyridoxine (**B**). The figures show the mean and standard error of the maternal serum levels with regards to the observed PUQE-score values. All three study groups were merged for this analysis.

In exploring the potential influence of maternal thyroxine or pyridoxine (vitamin B6) levels on the clinical course of emesis gravidarum, we calculated within-subject correlations between the PUQE-24 score and the before-mentioned serum levels. Our analysis revealed a strong positive and highly significant correlation between the PUQE-24 score and maternal thyroxine levels (r = 0.52, *p* < 0.001; Figure 3A). A moderate positive association was observed between the PUQE-24 score and maternal vitamin B6 serum levels (r = 0.33, *p* < 0.001; Figure 3B). However, it is important to note that this is an overall correlation, and there are relatively few patients with a PUQE-24 score > 8. In the higher value range, the correlations for both, thyroxine and pyridoxine reverses, but this is not reflected in the correlation coefficient, as only a small portion of the data falls within this range. Figure 3A,B, however, acknowledges this biphasic nature.

## 4. Discussion

In the presented prospective, randomized, double-blinded, placebo-controlled trial, we found that high maternal hCG levels but not histamine levels are associated with the clinical course of emesis gravidarum. The assessment of chewing gum for reducing the symptoms of emesis gravidarum found that neither a vitamin C-containing chewing gum nor a placebo chewing gum had the ability to improve symptoms of NVP. We also evaluated the influence of thyroxine and pyridoxine on the clinical course of NVP and observed that high levels of both thyroxine and vitamin B6 were associated with the course of emesis gravidarum.

The pathophysiology of emesis gravidarum is still poorly understood; however, it is widely accepted that NVP has a multifactorial pathogenesis [1,23]. The prevailing hypothesis implicates hCG as the central causative factor for NVP [1,23]. This theory is supported by the temporal correlation between the peak periods of NVP symptoms and hCG production, typically occurring around 9 to, at the latest, 16 weeks of gestation [1,24]. Furthermore, studies suggest that hCG levels tend to plateau or decline in tandem with the resolution of NVP symptoms [1,25]. Additionally, pregnant individuals with conditions associated with elevated hCG levels, such as molar pregnancies, multiple gestations or Down syndrome often experience more severe nausea and vomiting [5,26]. However, a systematic review including 35 studies conducted in 2014 by Niemeijer et al. [27] found very conflicting results with 18 studies that showed a significant association of high hCG levels and symptoms of NVP, 13 studies that did not observe such an association and 3 studies that found a correlation between lower levels of hCG in patients with emesis gravidarum. Our study revealed a highly significant correlation between elevated maternal hCG levels and the severity of NVP evaluated through the PUQE-24 score and thus supports the hypothesis that hCG is one of the central factors in the pathogenesis of emesis gravidarum. Additionally, our study found a significant reduction in PUQE-24 scores from baseline to both follow-up visits, which is consistent with the typical progression of NVP and aligns with the decreasing hCG levels over the course of pregnancy [1,5,23,25].

Linking pathogenesis to the treatment of NVP, antihistaminic agents, both with and without adjunctive pyridoxine, have demonstrated efficacy in treating NVP in several studies and are recommended as first-line therapy by several societies [1,23,28,29]. Their antiemetic effect is mediated via the vestibular nausea pathway by inhibiting histamine H1 receptors within the vomiting center and thereby interrupting communication with the chemoreceptor trigger zone [30]. The effect of antihistamines as treatment for NVP suggests a role of histamine in the pathogenesis of NVP. Historic studies described elevated blood, plasma and urine histamine levels as well as reduced DAO activity in patients with NVP [31,32]. However, these investigations could not find an association between histamine levels and the severity of NVP [31,32]. Our results confirm these historic observations since we could not identify any association between maternal histamine serum levels and the severity of NVP measured via the PUQE-24 score. Nevertheless, since increased levels of maternal blood histamine are linked to several pregnancy complications, such as pre-eclampsia, preterm labor, spontaneous abortion, and emesis gravidarum, and antihistamines have been proven effective for treatment, further investigations in this area are needed to identify the role of histamines in the aforementioned pregnancy complications [33].

In contrast, our research revealed a strong correlation between elevated serum DAO levels and lower PUQE-24 scores. As early as the 1940s and 1950s, various studies implied a potential role of DAO levels in early pregnancy as a marker for NVP [31,32]. However, the role of DAO in NVP is still underinvestigated and warrants future studies to define its precise involvement in emesis gravidarum.

With the assumption of an association between maternal histamine serum levels and the clinical course of emesis gravidarum at the time of study planning, we were seeking to find an easy-to-administer treatment option that is efficient in improving the symptoms of NVP. Vitamin C has long been used empirically to manage common colds, seasickness, and histamine intolerance [13,14]. A study by Hagel et al. [13] was able to prove that intravenous vitamin C infusion was reducing the histamine serum levels in allergic and non-allergic patients. Additionally, a randomized controlled pilot trial revealed that the use of chewing gums was not inferior to ondansetron for the treatment of postoperative nausea and vomiting (PONV) following laparoscopic or breast surgery among female patients [15]. The proposed mechanisms for its effect revolve around the concept of “sham feeding”, where chewing initiates increased gastrointestinal activity through cephalic vagal stimulation [34]. Based on these findings, we developed a chewing gum formulation containing vitamin C to treat NVP. However, our study revealed that neither the vitamin C-containing nor the placebo gum has an NVP-reducing effect. We attribute this lack of efficacy to our concurrent finding that maternal histamine levels do not influence the severity of NVP symptoms.

In regard to the first secondary aim of our study, it should be stated that the role of thyroid hormones in NVP has so far brought up conflicting findings [5,11,12,35]. A systematic review encompassing 34 studies reported that 65% of the included studies demonstrated decreased maternal TSH levels and 67% indicated increased thyroxine (fT4) levels in symptomatic patients [27]. In our study, we observed a clear association between elevated fT4 levels and the severity of NVP. However, given the heterogeneous nature of current research findings, further large-scale studies are required to define the exact role of thyroid hormones in the pathophysiology of emesis gravidarum.

The other secondary aim of our study was to investigate the role of pyridoxine (vitamin B6) in the clinical course of NVP. Pyridoxine alone or in combination with doxylamine has proven to be effective and safe as a treatment for NVP and is currently recommended as the first-line treatment according to several international guidelines [28,29,36]. Pyridoxine in its coenzyme state, is integral to cellular metabolism, facilitating the processing of amino acids, neurotransmitters, hormones, and glycogen. In NVP prevention, its effect is exerted by an inhibition of gene expression enhancement of steroid hormones [36,37]. Thereby, it is believed that the pyridoxine’s metabolite pyridoxal 5-phosphate (PLP) exhibits the antiemetic effect [37,38]. In previous research, higher levels of PLP were associated with an improvement of NVP symptoms [38]. Interestingly, our study revealed an increase in the severity of emesis gravidarum measured by the PUQE-24 score with rising pyridoxine levels. However, we did not measure the levels of the metabolites but only the overall pyridoxine level. Additionally, we observed a trend reversal at PUQE-24 values > 8, which was, however, not significant. Although, the effect of vitamin B6 as treatment has been known for many decades and is recommended as first-line treatment, the exact mechanism of pyridoxine and its metabolites in the pathophysiology of emesis gravidarum is poorly investigated and warrants further research in the future.

In recent years, research on NVP has increasingly emphasized genetic factors as a focus point of investigation. Studies about familial clustering and twins showed that daughters of mothers who experienced HG exhibit a threefold higher risk of developing HG themselves [39]. Moreover, sisters of HG-affected individuals face a 17-fold increased likelihood of experiencing HG during pregnancy [40]. In 2018, Fejzo et al. [41] discovered that the gene GDF15, which causes appetite loss, taste aversion and nausea in the brainstem, is associated with the severity of NVP [40]. In a follow-up study, they discovered that the GDF15 in the maternal serum is derived from the feto-placental unit and that maternal sensitivity to GDF15 is a major determinant in the course of NVP [42]. However, the relationship between hCG levels, estrogen, progesterone, thyroid hormones, pyridoxine and GDF15 levels has not been established up to date and merits further investigation. In the future, an interdisciplinary approach to emesis gravidarum is warranted, encompassing all contributing factors of this multifactorial disease, in order to identify appropriate treatment options and alleviate the burden of suffering from this common disease in early pregnancy.

The strengths of our study encompass the prospective, randomized, double-blinded design of our investigation and the broad array of maternal parameters that we evaluated. While numerous studies investigated individual parameters linked to the pathophysiology of emesis gravidarum, our contribution lies in the holistic evaluation of several maternal parameters within a single patient cohort. Additionally, we tested a novel treatment for NVP, which is easy to administer and has no adverse effects. Regrettably, our vitamin C-containing chewing gum did not yield significant improvement of NVP symptoms among our cohort. Our study also has several limitations. The cohort size of 126 pregnant women is relatively small for the evaluation of factors causing NVP in the maternal serum. However, despite this limitation, our findings revealed statistically significant differences within our cohort. Additionally, for the sake of the restrictions during the COVID-19 pandemic, we had to decrease the initially planned size of the study groups of 45 participants per group during the active phase of enrolment. Nevertheless, even with the adjusted numbers, our study maintained a robust 80% statistical power.

## 5. Conclusions

This study highlights the complexity of factors contributing to NVP, including hormonal and enzymatic influences. We have established a clear association between elevated maternal hCG levels and the severity of emesis gravidarum, supporting the understanding of hCG as a central causative factor in NVP. We also found a negative correlation between DAO levels and NVP symptoms and observed associations between increased maternal thyroxine and pyridoxine levels and the clinical course of emesis gravidarum. Maternal histamine levels, on the contrary, were not associated with the severity of NVP symptoms. The histamine decreasing capacity of vitamin C did not alleviate NVP symptoms when being administered as chewing gum in our in our prospective study setting. Given the multifactorial nature of this disease, with genetic, endocrine, and gastrointestinal factors involved in the pathogenesis, further large-scale studies are warranted to better understand the role of each factor and how they interact with each other in order to enable the development of targeted and effective treatments for NVP.

## Figures and Tables

**Table 1:** Maternal baseline characteristics at inclusion of the 111 enrolled participants.

Maternal Characteristic	Vitamin C Group (*n* = 41)	Placebo Group (*n* = 35)	No Treatment Group (*n* = 35)	*p*-Value
Mean Age (years)	33.2 (±6.1)	32.2 (±4.9)	32.2 (±5.2)	0.65
Gestational week at inclusion (weeks)	10 [9–11]	10 [9–11]	10 [9–11.5]	0.85
NVP onset (gestational week)	5.3 (±1.6)	6.0 (±1.9)	5.5 (±1.1)	0.15
PUQE-24 score at inclusion (points)	8 [7–10]	9 [7–10]	8 [7–9]	0.25
Nulliparous women	12 (29,3%)	9 (25,7%)	9 (25,7%)	0.43
Nicotine abuse	6 (14.6%)	4 (11.4%)	4 (11.4%)	0.89
NVP in prior pregnancy	16 (39.0%)	15 (42.9%)	17 (48.6%)	0.70
HG in prior pregnancy	0 (0.0%)	2 (5.7%)	1 (2.9%)	0.31
Thyroid disease	7 (17.1%)	5 (14.3%)	6 (17.1%)	0.93
Any chronic disease	15 (36.6%)	19 (54.3%)	17 (48.6%)	0.28

Data are presented as number (percentage), mean (±standard deviation), or median [interquartile range]. PUQE-24, pregnancy-unique quantification of emesis and nausea; NVP, nausea and vomiting pregnancy; HG, hyperemesis gravidarum. *p* values for the between-groups comparison of mean age and NVP onset were calculated using analysis of variance. *p* values for the comparison of gestational week at inclusion and PUQE-24 score at inclusion were calculated using Kruskal–Wallis tests. *p* values for the comparisons regarding categorical variables were calculated using Pearson’s Chi-squared tests.

## Data Availability

The raw data supporting the conclusions of this article will be made available by the corresponding author upon reasonable request.

## Supplementary Materials

The following supporting information can be downloaded at: https://www.mdpi.com/article/10.3390/jcm13175099/s1, Figure S1: PUQE-24 Form; Figure S2: Study procedures executed at each study visit (inclusion, follow-up I and follow-up II); Figure S3: Study inclusion of a total of 126 pregnant women. Out of these, 15 women had to be excluded from the study during the first or second follow-up visit. The data of the remaining 111 women were available for statistical analyses. This figure shows inclusion, randomization and drop-outs in the study, placebo and control groups, respectively.

## Abbreviations

NVP	Nausea and vomiting in pregnancy
hCG	Human chorionic gonadotropin
DAO	Diamine oxidase
PUQE-24	Pregnancy-Unique Quantification of Emesis and Nausea
HG	Hyperemesis gravidarum
fT4	Thyroxine
TSH	Thyrotropin
