# Supplementary material for: The Role of hCG and Histamine in Emesis Gravidarum and Use of a Chewing Gum Containing Vitamin C as a Treatment Option: A Double-Blinded, Randomized, Controlled Trial
Source: J Clin Med. 2024 Aug 28;13(17):5099. doi: 10.3390/jcm13175099 (PMC11396101; doi:10.3390/jcm13175099)
Supplement: Supplementary file 1 [file jcm-13-05099-s001.zip › jcm-3152693-supplementary.pdf]

The role of hCG and histamine in emesis gravidarum and use of a chewing gum containing vitamin C as a treatment option: a double-blinded, randomized, controlled trial

Supporting information

Figure S1:

PUQE (pregnancy-unique quantification of emesis and nausea)-24 Form*

Please mark the answer that best describes your situation during the last 24 hours!

1) On an average day, for how long do you feel nauseated or sick to your stomach?

**> 6 hours**<br>5 points	**4 - 6 hours**<br>4 points	**2 - 3 hours**<br>3 points	**≤ 1 hours**<br>2 points	**Not at all**<br>1 points

2) On an average day, how many do you vomit or throw up?

**≥ 7 times**<br>5 points	**5 – 6 times**<br>4 points	**3 – 4 times**<br>3 points	**1 – 2 times**<br>2 points	**Not at all**<br>1 points

3) On an average day, how many times do you have had retching or dry heaves without bringing anything up?

**≥ 7 times**<br>5 points	**5 – 6 times**<br>4 points	**3 – 4 times**<br>3 points	**1 – 2 times**<br>2 points	**Not at all**<br>1 points

Additional question (Quality of Life): On a scale from 0 (the worst possible) to 10 (as good as before pregnancy), how would you rate your general well-being?

Quality of Life - Score: _____________ (0 – 10 points)

PUQE-24 Score: _____________ (3 – 15 points)

PUQE-24 Form modified from: Koren G, Boskovic R, Hard M, Maltepe C, Navioz Y, Einarson A. Motherisk-PUQE (pregnancy-unique quantification of emesis and nausea) scoring system for nausea and vomiting of pregnancy. American journal of obstetrics and gynecology 2002; 186(5 Suppl Understanding): S228-31. *With permission of the authors/publisher.*

*** For the study purpose, a German translation of this form was used.**

Figure S2:

Study procedures executed at each study visit (inclusion, follow-up I and follow-up II).

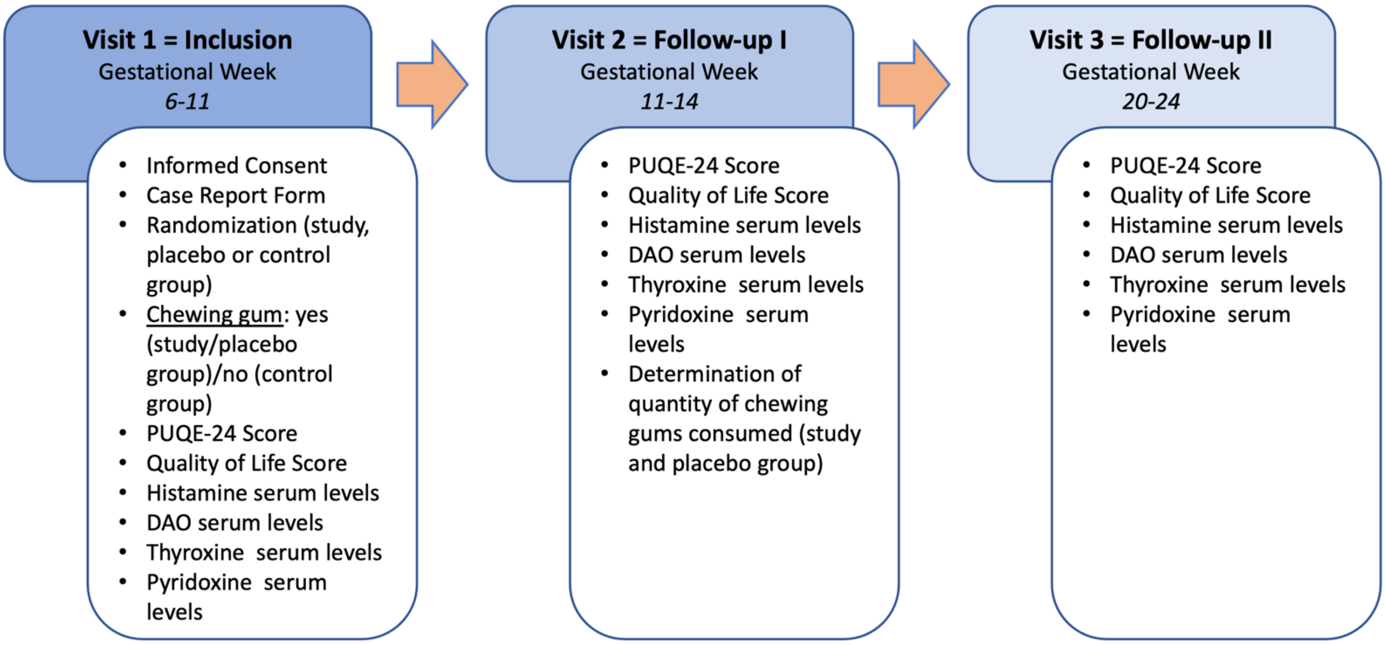

PUQE, pregnancy-unique quantification of emesis and nausea; DAO, diamine oxidase

Figure S3:

Study inclusion of a total of 126 pregnant women. Out of these, 15 women had to be excluded from the study during the first or second follow-up visit. The data of the remaining 111 women were available for statistical analyses. This figure shows inclusion, randomization and drop-outs in the study, placebo and control groups, respectively.

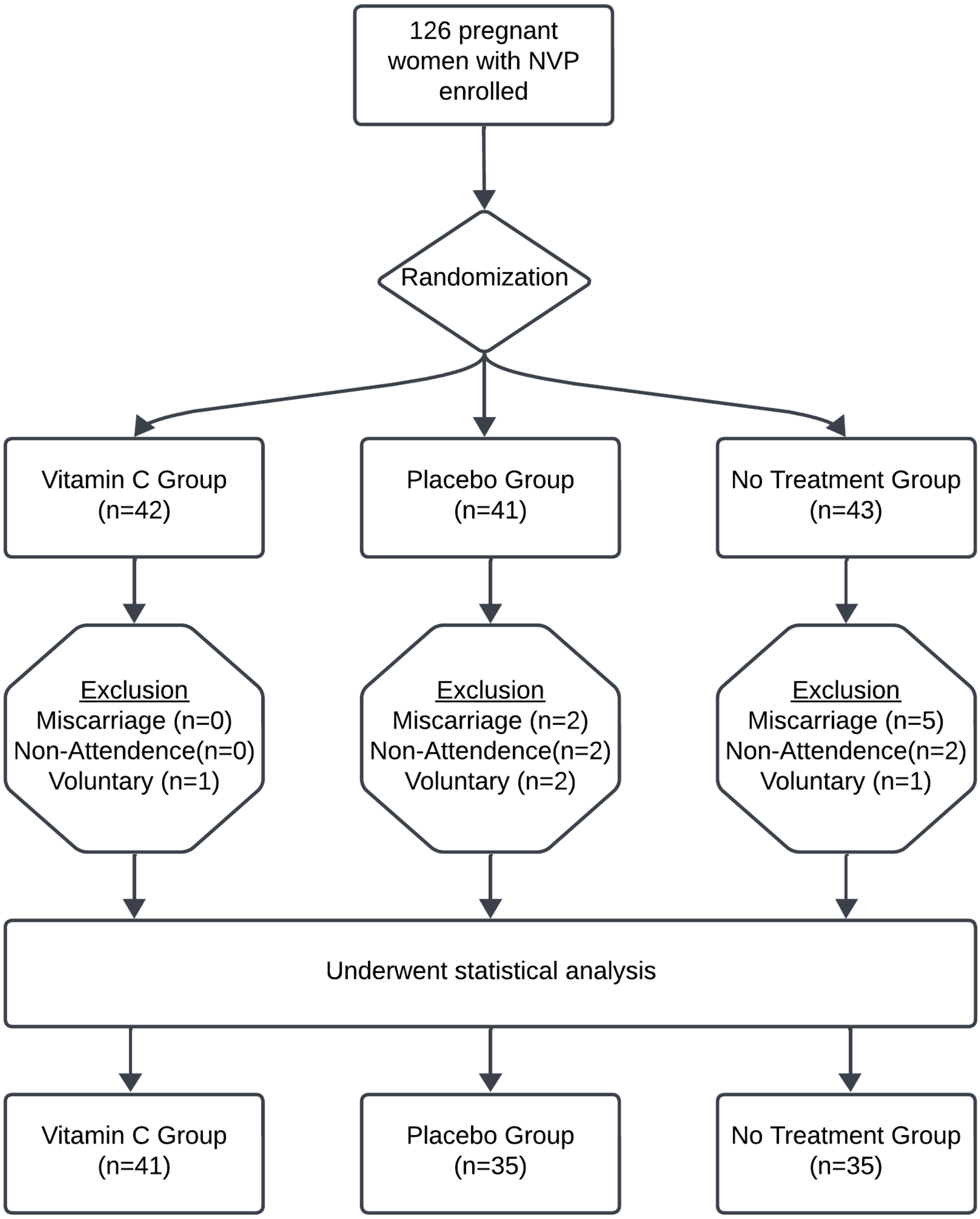

NVP, nausea and vomiting in pregnancy
